# A case report: Hemophagocytic lymphohistiocytosis and thrombotic thrombocytopenic purpura in an otherwise healthy woman

**DOI:** 10.1097/MD.0000000000033803

**Published:** 2023-05-17

**Authors:** Yuanyuan Li, Wenqiang Li, Zhen Li, Fubing Ma, Baocai Xu

**Affiliations:** a Department of ICU, Jining No.1 People’s Hospital, Jining, China; b Department of Urology, Jining No.1 People’s Hospital, Jining, China.

**Keywords:** ADAMTS13, case report, hemophagocytic lymphohistiocytosis, plasma exchange, thrombotic thrombocytopenic purpura

## Abstract

Thrombotic thrombocytopenic purpura (TTP) with hemophagocytic lymphohistiocytosis (HLH) is very rare, and both of these rare blood diseases have high mortality. There have been few reports of 2 diseases being combined at the same time. We provide a rare case with a clear diagnosis, prolonging the patient's survival through aggressive treatment, providing clinicians with our experience in early diagnosis and early treatment of this disease.

**Patient concerns:**

A 56-year-old woman presented with a 1-month history of fever.

**Diagnoses:**

She was diagnosed with HLH due to elevated levels of ferritin and lactase dehydrogenase, which were confirmed by the presence of hemophagocytosis in the bone marrow. TTP was diagnosed based on the presence of symptoms characteristic of TTP and significantly low levels of ADAMTS13 (a disintegrin and metalloprotease with thrombospondin type 1 repeats, member 13).

**Interventions:**

Systemic corticosteroids and plasma exchange using 2 L of virus-inactivated frozen plasma per day were initiated as specific treatment.

**Outcomes:**

The patient’s consciousness improved posttreatment and platelets also increase gradually. In a follow-up after 1 month, the patient was generally well and without specific discomfort.

**Lessons:**

HLH patients themselves can have a significant reduction in platelet, as with TTP, it is very easy to misdiagnose or delay the diagnosis. How to diagnose early, actively find the primary disease, and treat it is crucial to improve the prognosis of HLH.

## 1. Introduction

Hemophagocytic lymphohistiocytosis (HLH) is a life-threatening syndrome characterized by an unchecked and persistent activation of cytotoxic T lymphocytes and natural killer cells. The mortality rate of HLH among patients in the intensive care unit is high, and its overall mortality reaches up to 57.8%.^[1]^ On the other hand, thrombotic thrombocytopenic purpura (TTP) is a fatal clinical syndrome, primarily caused by autoantibodies against von Willebrand factor (VWF)-cleaving metalloprotease a disintegrin and metalloprotease with thrombospondin type 1 repeats, member 13 (ADAMTS13). Since HLH may cause a significant reduction in platelet count and neurological changes, it is often misdiagnosed in patients also presenting with TTP. Prompt diagnosis and targeted treatment are essential to improve the prognosis of patients with HLH and TTP. We report a case of a patient presenting with fever. She was diagnosed with HLH due to elevated levels of ferritin and lactase dehydrogenase, which were confirmed by the presence of hemophagocytosis in the bone marrow. However, hemophagocytosis may also be observed in patients with neuropsychiatric symptoms. The patient was initially believed to have TTP. TTP was diagnosed based on the presence of symptoms characteristic of TTP and significantly low levels of ADAMTS13.

## 2. Case presentation

An otherwise healthy woman presented with a 1-month history of fever. The patient had no known allergies or a family history of any hereditary diseases. She also had cough, fatigue, and loss of appetite. She was initially managed with anti-infective fluids, but her fever did not improve. Initial laboratory studies performed at a local grade A tertiary hospital revealed elevated serum ferritin levels: 21,629.82 (13–50) ng/mL. Ultrasound revealed multiple hypoechoic nodules in the bilateral axilla, subclavian, neck, and supraclavicular regions. Enlarged lymph nodes were considered. Increased cytoplasmic granules in the granulocyte part of bone marrow, indicative of possible infection, were observed on bone marrow aspiration. The patient was diagnosed with HLH. Tumors were not observed in the neck, chest, and abdominal positron emission tomography-computed tomography. Furthermore, her bone marrow culture and blood tests for connective tissue disease were all negative. During her entire hospitalization, persistent fever was observed, prompting admission to Jining First People’s Hospital.

On admission, the patient had a temperature of 38.1°C, blood pressure of 94/64 mm Hg, pulse rate of 76 beats/minutes, and respiratory rate of 19 breaths/minutes. On physical examination, bilaterally enlarged lymph nodes in the neck, axilla, and groin were observed. Her initial laboratory studies are summarized Table 1. She was diagnosed with HLH, and was administered systemic corticosteroids. A therapeutic dose of 400 mL leucocyte-depleted suspended red blood cells and platelet concentrate was given. She was also given empiric broad-spectrum antibiotics.

**Table 1:** Laboratory exams.

Laboratory exams	Patient	Reference values
WBC (10*9/L)	7.08	3.5–9.5
HBG (g/L)	73	115–150
PLT (10*9/L)	12	125–350
RET (%)	4.11	
FRCs (%)	4	
LDH (U/L)	1728	120–246
TBIL (Umol/L)	93.1	0–23
IBIL (Umol/L)	74.8	0–15.2
BUN (mmol/L)	7.05	2.5–6.1
Cr (Umol/L)	44	46–92
FERR (ng/mL)	>2000	13–150
PRO	1+	

BUN = Blood urea nitrogen, Cr = Creatinine, FERR = Ferritin, FRCs = Fragmented red cells, HBG = Hemoglobin, IBIL = Indirect bilirubin, LDH = Lactate dehydrogenase, PLT = Platelet, PRO = Urine protein, RET = Reticulocyte, TBIL = Total bilirubin, WBC = White blood cell.

**Figure 1.**
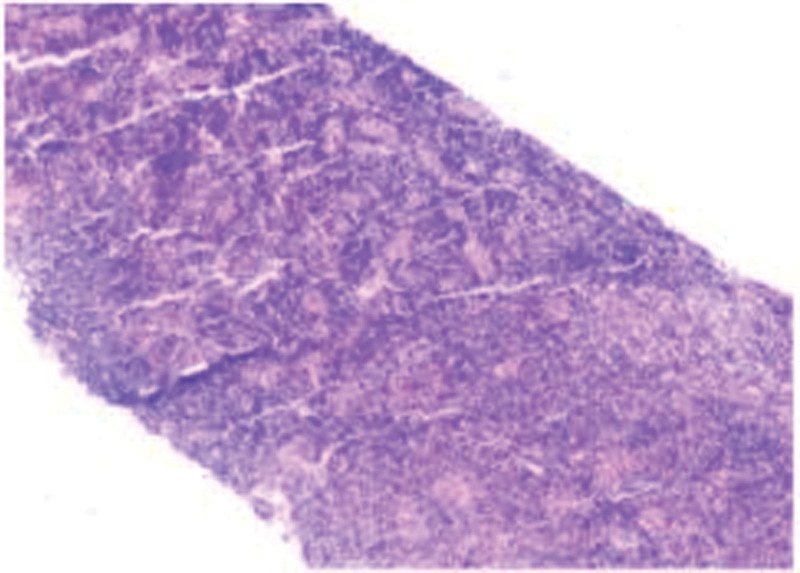
Lymph node biopsy. A hypoechoic nodule in the right inguinal area was observed on pathological examination. Reactive lymphoid hyperplasia was considered, and a follow-up review was recommended. Immunohistochemical: Bcl-2 (part +), CD10 (−), CD20 (part +), CD3 (part +), CD5 (part +), CD79a (part +), CD43 (part +), CD21 (FDC +), CD23 (FDC +), and K1-67 (+) about 5%–10%; In situ hybridization: EBER (−).

**Figure 2.**
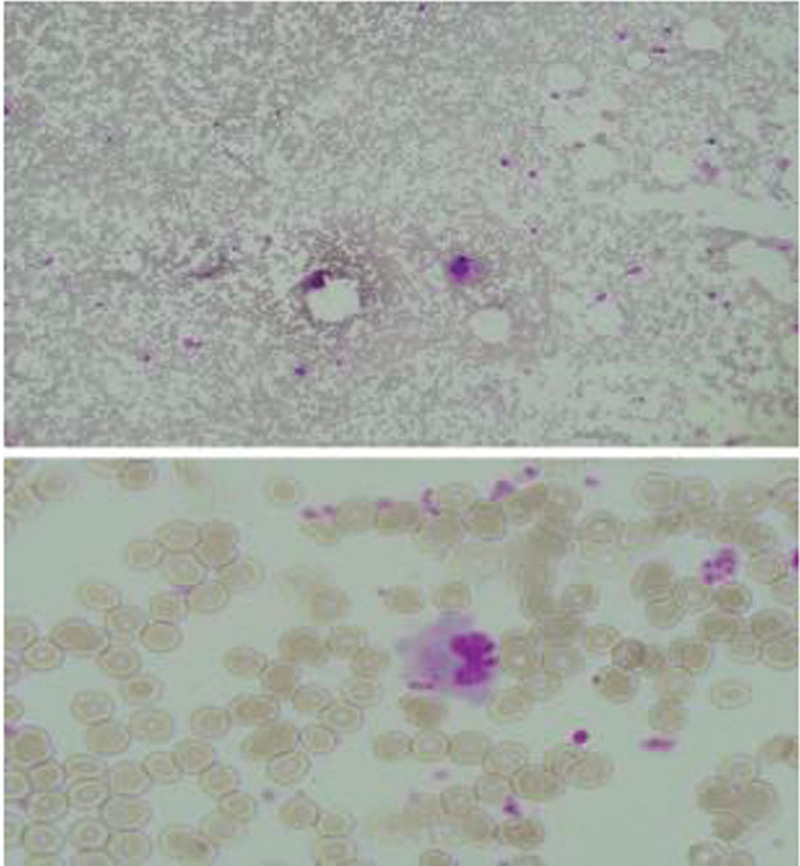
Bone marrow cell examination. Occasional bone marrow-like phagocytic cells were observed.

The next day, the patient had a low platelet count of 5 × 10^9^/L. She entered a state of sudden-onset convulsions, poor mental state, inability to cooperate, and dysphoria. The results of her brain computed tomography scan were unremarkable. Since TTP was likely, the patient was started on plasma exchange (PEX) therapy. She received a total of 17 cycles of PEX. In the first 12 days, PEX, using approximately 2 L of virus-inactivated frozen plasma per day, was performed. No platelet transfusions were administered during this period. The patient responded to therapy, with the patient’s mental state improve, and she could answer simple questions. Her platelet c with the patient’s mental state improvement, and she could answer simple questions. Her platelet ount gradually increased to 49 × 10^9^/L. After the 12^th^ cycle of PEX, her platelet count increased to 117 × 10^9^/L. Since her temperature was not higher than 37.5 °C, only etoposide was administered. The patient underwent lymph node biopsy and bone marrow aspiration (Figs. 1 and 2). Additional laboratory examination revealed increased sCD25 (alpha chain of the soluble interleukin-2 receptor, sIL-2Rα) levels: 1783.20 IU/mL (13.1–43.7), decreased ADAMTS13 activity: 1.82% (42.16–126.37%), and negative ADAMTS13 autoantibodies. The patient’s mental state improved, while the platelet gradually increased, and the family members agreed with our treatment.

On the 8th day of hospitalization, her white blood cell count decreased to 2.3 × 10^9^/L. She was given human granulocyte stimulating factor. On the 14^th^ day of hospitalization, her fever rose to 39.2 °C. She was given multiple antibiotics, including imipenem and cilastatin sodium, vancomycin hydrochloride, and voriconazole to manage her infection. No bacteria was noted on sputum culture, blood culture, catheter blood culture, and catheter tip culture.

On the 25^th^ day of hospitalization, the patient went home against medical advice due to financial and compliance issues. Prior to discharge, her temperature was still 39 °C, and her white blood cell count was still 0.33 × 10^9^/L. On hospital discharge, the patient was alert and had a normal platelet count.

## 3. Discussion

We report a case of an otherwise healthy patient diagnosed with HLH. The diagnosis of HLH was made due to elevated ferritin and lactase dehydrogenase levels, which were confirmed by hemophagocytosis in the bone marrow.^[2]^ TTP was also diagnosed in the patient. The diagnosis of TTP was made based on the standard clinical pentad criteria, and confirmed using ADAMTS13 assays. The incidence ratio of TTP among women and men is approximately in the ratio 2:1, with a peak age of onset of 30 to 40 years. Women often present with early disease flares in pregnancy.^[3]^ We present a rare case of a 56-year-old woman with both HLH and TTP.

HLH is a rare and fatal hyperinflammatory syndrome that mimics sepsis in critically-ill patients. Diagnosis relies on the HLH-2004 criteria and H-Score, both of which are used in pediatric or adult noncritically-ill patients, respectively. Both the HLH-2004 criteria and H-Score proved to be of good diagnostic accuracy for HLH diagnosis in critically-ill patients.^[4]^ According to its etiology, the disease can be divided into 2 categories. Primary or familial HLH is primarily seen in children under 2 years of age. On the other hand, acquired HLH is usually caused by infections, tumors, and rheumatic immune diseases, and is most commonly seen in adults. HLH associated with malignant tumors is more common. Lymphoma-associated HLH accounts for approximately 15% to 50% of acquired HLH. Most scholars believe that it is related to the abnormality of *PRFl* gene encoding perforin.^[5]^ HLH causes hyperactivity due to increased monocytes and macrophages, which can initiate the activation of a cytokine storm. The most common cytokines are interleukin (IL)-6, tumor necrosis factor α, and interferon γ.^[6,7]^

TTP is an uncommon life-threatening disease that requires prompt diagnosis and initiation of therapeutic PEX. However, diagnosis is often challenging due to its atypical signs and symptoms that resemble other conditions. Measurements of ADAMTS13 activity, ADAMTS13 inhibitor, and ADAMTS13 autoantibodies are useful in diagnosing TTP, guiding its therapy, and predicting possible relapse.^[8,9]^ ADAMTS13 is an essential component in the pathophysiology of TTP.^[10]^ ADAMTS13 can stimulate complement pathways and inflammatory cytokines, such as IL-6 and IL-10, and autoreactive B and T cells.^[11]^ Regarding the immune abnormalities in TTP and HLH, overlapping immune dysregulation is the principal association between these 2 diseases.

TTP management consists of 3 main strategies: therapies to supply ADAMTS13, immunomodulators that target antiADAMTS13 autoantibodies, and antiVWF drugs that prevent the formation of platelet-rich microthrombi.^[12]^ The standard treatment of TTP is PEX, which removes antiADAMTS13 neutralizing autoantibodies and harmful cytokines from the circulation and replenishes ADAMTS13.^[13]^ Although recovery in patients with acute attacks of TTP is possible, there is an increased risk of repeated acute attacks.^[14]^

Although multiple studies have been conducted, the exact cause of HLH remains unclear. Decreased white blood cells in patients may be related to uncontrolled HLH. Moreover, an infection may further aggravate the disease. Early and extensive investigation in the diagnosis of HLH along with further insight in its pathophysiology will decrease complications and improve the survival and quality of life in patients in remission from TTP.

## 4. Conclusion

HLH patients themselves can have a significant reduction in Platelet, as with TTP, it is very easy to misdiagnose or delay the diagnosis. How to diagnose early, actively find the primary disease and treat it is crucial to improve the prognosis of HLH.

## Acknowledgments

The authors thank the leaders of the Department of Critical Care Medicine of Jining First People’s Hospital, for its assistance.

## Author contributions

**Conceptualization:** Wenqiang Li.

**Data curation:** Baocai Xu.

**Investigation:** Zhen Li, Fubing Ma.

**Writing – original draft:** Yuanyuan Li.

## References

[1] Knaak C Schuster FS Nyvlt P. Treatment and mortality of hemophagocytic lymphohistiocytosis in adult critically ill patients: a systematic review with pooled analysis. Crit Care Med. 2020;48:e1137–46. 32947471 10.1097/CCM.0000000000004581

[2] Henter JI Horne A Aricó M. HLH-2004: diagnostic and therapeutic guidelines for hemophagocytic lymphohistiocytosis. Pediatr Blood Cancer. 2007;48:124–31. 16937360 10.1002/pbc.21039

[3] Miller DP Kaye JA Shea K. Incidence of thrombotic thrombocytopenic purpura hemolytic uremic syndrome. Epidemiology. 2014;15:208–15. 10.1097/01.ede.0000113273.14807.53 15127914

[4] Knaak C Nyvlt P Schuster FS. Hemophagocytic lymphohistiocytosis in critically ill patients: diagnostic reliability of HLH-2004 criteria and HScore. Crit Care. 2020;24:244. 32448380 10.1186/s13054-020-02941-3 PMC7245825

[5] Janka GE. Hemophagocytic syndromes. Blood Rev. 2007;21:245–53. 17590250 10.1016/j.blre.2007.05.001

[6] Rivière S Galicier L Coppo P. Reactive hemophagocytic syndrome in adults: a retrospective analysis of 162 patients. Am J Med. 2014;127:1118–25. 24835040 10.1016/j.amjmed.2014.04.034

[7] Brisse E Matthys P Wouters CH. Understanding the spectrum of haemophagocytic lymphohistiocytosis: update on diagnostic challenges and therapeutic options. Br J Haematol. 2016;174:175–87. 27292929 10.1111/bjh.14144

[8] Rogers HJ Allen C Lichtin AE. Thrombotic thrombocytopenic purpura: the role of ADAMTS13. Cleve Clin J Med. 2016;83:597–603. 27505881 10.3949/ccjm.83a.15009

[9] Mariotte E Azoulay E Galicier L. Epidemiology and pathophysiology of adulthood-onset thrombotic microangiopathy with severe ADAMTS13 deficiency (thrombotic thrombocytopenic purpura): a cross-sectional analysis of the French national registry for thrombotic microangiopathy. Lancet Haematol. 2016;3:e237–45. 27132698 10.1016/S2352-3026(16)30018-7

[10] Sadler JE. Pathophysiology of thrombotic thrombocytopenic purpura. Blood. 2017;130:1181–8. 28768626 10.1182/blood-2017-04-636431 PMC5606001

[11] Westwood JP Langley K Heelas E. Complement and cytokine response in acute thrombotic thrombocytopenic purpura. Br J Haematol. 2014;164:858–66. 24372446 10.1111/bjh.12707 PMC4155869

[12] Joly BS Vanhoorelbeke K Veyradier A. Understanding therapeutic targets in thrombotic thrombocytopenic purpura. Intensive Care Med. 2017;43:1398–400. 28116452 10.1007/s00134-016-4662-3

[13] Joly BS Coppo P Veyradier A. Thrombotic thrombocytopenic purpura. Blood. 2017;129:2836–46. 28416507 10.1182/blood-2016-10-709857

[14] Chaturvedi S Abbas H McCrae KR. Increased morbidity during long-term follow-up of survivors of thrombotic thrombocytopenic purpura. Am J Hematol. 2015;90:E208E208–E208. 26228638 10.1002/ajh.24138 PMC4579009

